# Impact of Ambient Air Pollution Exposure on Long COVID-19 Symptoms: A Cohort Study within the Saudi Arabian Population

**DOI:** 10.3390/idr15050060

**Published:** 2023-10-19

**Authors:** Saleh A. K. Saleh, Heba M. Adly

**Affiliations:** 1 Biochemistry Department, College of Medicine, Umm Al-Qura University, Makkah 21955, Saudi Arabia; saabdrabou@uqu.edu.sa; 2 Oncology Diagnostic Unit, College of Medicine, Ain Shams University, Cairo 11435, Egypt; 3 Community Medicine and Pilgrims Healthcare Department, College of Medicine, Umm Al-Qura University, Makkah 21955, Saudi Arabia

**Keywords:** COVID-19, long COVID, air pollutants, PM10, PM2.5, post symptoms

## Abstract

Evidence suggests that air pollution, specifically the particulate matters PM2.5 and PM10, plays a key role in exacerbating the risk of prolonged symptoms following COVID-19 infection. Aim: This study endeavors to elucidate the potential interaction between chronic air pollution exposure and the manifestation of long COVID symptoms within a cohort based in Makkah, Saudi Arabia. Methods: Participants included residents from the Makkah region who had recovered from COVID-19 between 2022 and 2023. A comprehensive questionnaire was utilized to gather detailed demographic data and assess the persistent symptoms seen during the post-COVID period. To gauge the environmental exposure to potential risk factors, air sampling for PM10 and PM2.5 was systematically conducted in various locations in Makkah over a year. Results: Significant positive associations were found between PM2.5 and PM10 exposure and long COVID. Furthermore, specific symptom analysis revealed a significant association between air pollution and shortness of breath (for PM2.5). Only PM2.5 exposure remained statistically significant (RR = 1.32, 95% CI: 1.05, 1.67). In contrast, the association with PM10 remained on the cusp of significance, with an RR of 1.27 (95% CI: 1.00, 1.61). Conclusion: This study highlights the importance of reducing air pollution levels to mitigate the long-term health consequences of COVID-19.

## 1. Introduction

The COVID-19 pandemic has seen a growing concern regarding persistent symptoms experienced by individuals after recovering from SARS-CoV-2 infection, a condition now commonly referred to as long COVID. These symptoms can include fatigue, dyspnea, cough, and impaired smell and taste, among others, and are capable of lingering for several months after the acute infection [1,2]. Identifying potential risk factors for long COVID is crucial to understanding the disease and developing appropriate management strategies [3]. Recently, there has been interest in investigating the association between air pollution exposure and the development of long COVID symptoms [4].

Furthermore, an analysis using data from the COVID Symptom Study app found that higher levels of air pollution, particularly PM2.5 and nitrogen oxides (NOx), were associated with an increased risk of long COVID symptoms, including fatigue, a cough, and anosmia [5]. Similarly, a study by in the United States observed a significant association between exposure to PM2.5 and increased odds of long COVID symptoms, particularly fatigue, shortness of breath, and impaired smell and taste [6]. The association between air pollution exposure and long COVID symptoms can be attributed to several mechanisms [7]. Air pollutants, such as PM2.5 and NO_2_, have been shown to induce oxidative stress, inflammation, and respiratory damage, each of which can exacerbate the effects of SARS-CoV-2 infection and prolong the recovery process [8,9]. Furthermore, air pollution can impair immune function and respiratory health, making individuals more susceptible to persistent symptoms following COVID-19 [10].

The available evidence suggests a potential association between air pollution exposure and the development of long COVID symptoms [11]. Long-term exposure to air pollutants, such as PM2.5 and NO_2_, may contribute to the persistence and severity of symptoms following SARS-CoV-2 infection [12]. A study from Brazil found an association between exposure to ambient particulate matter with a diameter ≤2.5 μm (PM2.5) and a higher risk of dyspnea and fatigue among hospitalized adult patients [13]. Moreover, the importance of the timing of air pollution exposure, such as early life exposure when the lungs are more susceptible or exposure later in life, is poorly understood [14].

Air quality has been linked to the transmission of coronavirus, with studies suggesting that environmental pollutants cause there to be higher risk of COVID-19 occurrence, disease intensity, and mortality rates [15,16,17]. Moreover, the presence of pollutants in the air may elevate the likelihood of health complications connected to persistent COVID symptoms [18]. The reasons and patterns behind prolonged COVID symptoms are still not well researched, with sparse empirical data available [19].

However, it is important to note that the existing evidence on the association between air pollution and long COVID symptoms is still limited, and that further research is needed to establish a causal relationship between the factors and elucidate the underlying mechanisms at work [20,21].

This study seeks to analyze the relationship between sustained exposure to air contaminants, measured at population residential areas over distinct periods, and the emergence of long COVID within a demographic cohort in Saudi Arabia.

## 2. Materials and Methods

### 2.1. Study Design

This research examined individuals who recuperated from COVID-19 between 2022 and 2023. Only individuals with a confirmed diagnosis of COVID-19, as evidenced by a positive SARS-CoV-2 PCR test result from accredited testing facilities, were considered. This criterion ensured the authenticity of the COVID-19 infection among participants. Individuals who had been hospitalized due to severe COVID-19 symptoms were noted but not excluded, allowing for the study to capture a range of disease severities. However, we excluded those with critical comorbidities or those who were immunocompromised in order to prevent potential confounding. To qualify, participants were required to have fully recovered from their acute COVID-19 symptoms for at least three months, ensuring that the study was genuinely capturing the persistence of long COVID symptoms rather than acute post-infection symptoms. Individuals who had experienced more than one confirmed episode of COVID-19 were excluded to ensure clarity in associating air pollution exposure with symptom persistence from a single infection event. To gauge the prevalence of lingering COVID symptoms, we devised a survey to probe their current health and any ongoing post-COVID symptoms. Between January and March 2023, this survey was propagated across various digital media platforms. All respondents were mandated to give their informed consent before participating. The investigative protocol was greenlit by the Ethical Committee Board at the Faculty of Medicine, Umm Al-Qura University, under the reference (Approval no. HAPO-02-K-012-2022-01-902), thus adhering to the standards set by the Saudi National Committee for Bioethics HABO-02-K-012.

The survey structure involved the collection of fundamental details like age, gender, presence of long-standing health conditions, and previous hospital admissions (covering the severity of the illness, length of stay, requirement for breathing aids, and ICU admissions). Information about the duration since the start of symptoms and the persistence of specific symptoms organized by bodily functions was also sought. Respondents shared their experiences during the acute phase of COVID-19. The gravity of their COVID-19 condition was categorized into mild-to-moderate, severe, or critical, with assessment thus aligned with WHO’s guidelines for COVID-19 [22]. Symptoms were organized into categories: general (such as fatigue and muscle pain), respiratory (like chest discomfort and cough), cardiovascular (like heart flutter), neurologic/psychological (including headaches, excessive sleep, mood disorders), skin-related (like hair loss), and digestive (diarrhea, constipation). 

### 2.2. Air Particulate Matter (PM10 and PM2.5 Sampling)

**Figure 1:**
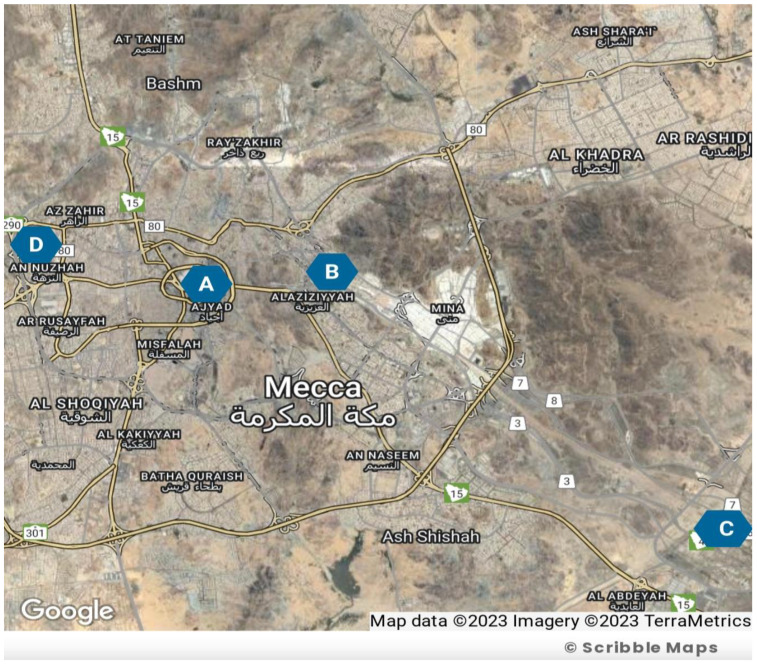
Sampling site location map adapted from Map Data, TerraMetrics 2023; Blue circles show site locations as follows: Circle A: Al-Haram; Circle B: Al-Aziziyah; Circle C: Arafat; Circle D: Al-Nuzhah.

In this study, PM10 and PM2.5 sampling collections were conducted in various areas of Makkah, including Al-Haram, Arafat, Al-Aziziyah, and Al-Nuzhah, as illustrated in Figure 1. The sampling period lasted for one year, starting from March 2022 and ending in March 2023. Table 1 shows the sampling location characteristics.

A 24 h air sampling approach was employed throughout all seasons for one year. A mini volume sampler (Airmetrics, USA) was utilized at a height of 10 meters, with a flow rate of 16.6 liters per minute. The air samples were collected on a 47 mm Teflon filter in accordance with the USEPA standard technique—USEPA-Method 29/2000 [23]. Weekly sampling was conducted, and each collected filter was carefully placed on a clean Petri dish. The weight of the filters was measured before and after sampling using a microbalance (CITIZEN, Torrance, CA, USA). The Teflon filters were conditioned in a dry locker, and their balance and conditioning were maintained at a temperature of 35–40 °C with a humidity range of 60–70%. The calibration frequency of a mini volume sampler (Airmetrics, Springfield, OR, USA) was assessed before the commencement of the study, and then quarterly throughout the sampling period. A primary standard flow calibrator, traceable to NIST standards, was used to calibrate the sampler. The sampler’s flow rate was set at 16.6 liters per minute, as per our study’s requirement. A side-by-side comparison with the primary standard was performed. Any deviation greater than 5% from the standard was adjusted. Calibration history was maintained, recording the date, initial and final flow rates, and any adjustments made.

For PM2.5 sampling, the same procedure was followed. However, in this case, Whatman 2.0 μm pore-size PTFE 46.2 mm filters were used, along with an Al cyclone separator with a cut-off size of 2.5 µm. The flow rate of the sampler was set at 16.67 ± 0.84 L/min, which was an optimal level for the PM2.5 sampling size.

### 2.3. Statistical Analysis

In our investigation, we used Poisson regression to assess the link between exposure levels of PM2.5 and PM10 air pollutants and the occurrence of long COVID symptoms, as reported by participants. This method is ideal for analyzing count outcomes due to its assumption that the outcome adheres to a Poisson distribution, where the variance matches the mean. Using this approach, we were able to determine the rate of occurrence of long COVID symptoms for each incremental increase in the exposure to the air pollutants, where the results are represented as rate ratios (RRs). We also reviewed our model for potential overdispersion; in instances where this was observed, we considered alternative models, such as the negative binomial regression All statistical analyses were conducted using SPSS software (Version 25.0, IBM Corp., Armonk, NY, USA). Continuous variables with a non-normal distribution were described as either mean ± SD or medians with interquartile ranges (25th–75th percentile). Categorical variables were presented as frequencies and percentages. For data that adhered to a normal distribution, we employed a *t* test for continuous variables and the χ^2^ test for categorical variables under parametric conditions. A *p* value below 0.05 was set as the threshold for statistical significance. For non-normal data, we employed non-parametric tests. The Mann–Whitney U test was used instead of the independent *t* test or ANOVA, respectively. Non-parametric tests make fewer assumptions about the data distribution and are more robust against violations of normality.

For assessing the association between persistent COVID-19 symptoms and various covariates, we employed a logistic regression model. Variables such as age, gender, comorbidities, and others were included as covariates. On the other hand, to determine the relationship between PM2.5/PM10 exposure and the frequency of long COVID symptoms, a Poisson regression model was utilized. In this analysis, the rate ratios of long COVID symptoms in relation to PM exposure levels were calculated, adjusting for potential confounders

In our study, we employed one-way sensitivity analysis to assess the robustness of our model outcomes. This method involves individually varying one input parameter at a time from its base case value, keeping all other parameters constant, in order to determine its impact on the model outcome. By isolating each variable in this manner, we were able to identify the parameters that had the most significant influence on our results and understand the range within which our conclusions remain valid. This approach provided a clearer picture of the individual uncertainties associated with each parameter and allowed for us to ascertain the stability of our primary findings against variations in specific inputs.

## 3. Results

### 3.1. Participants Demographic Characteristics

**Figure 2:**
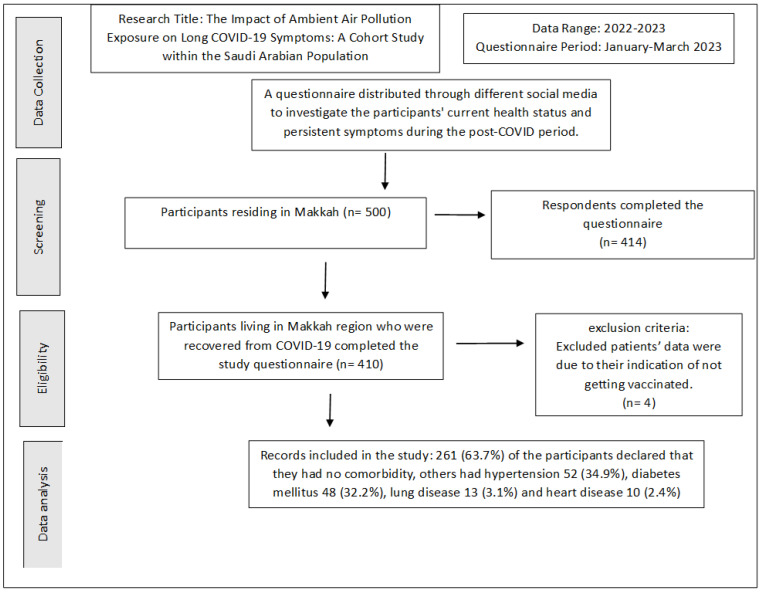
Study inclusion criteria.

**Figure 3:**
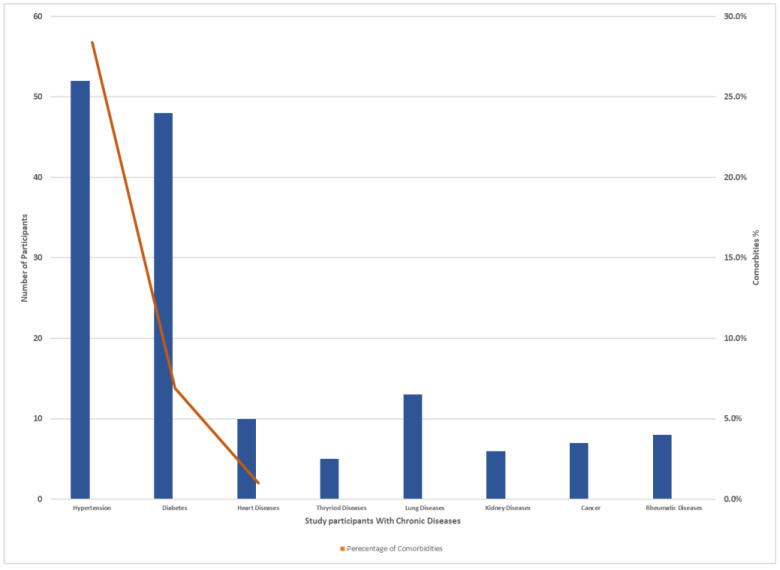
Number of chronic diseases and comorbidities percent distribution among study participants.

The study questionnaire was distributed among 500 individuals residing in Makkah, and the residents were distributed across the selected study locations (Al-Haram, Arafat, Aziziyah, and Al Nuzhah). A total of 414 respondents completed the questionnaires. Four individuals (0.9%) were excluded from the analysis as they indicated that they had not received vaccination. In total, 410 participants living in the Makkah region who recovered from COVID-19 completed the study questionnaire. Of the total participants, 204 (49.8%) were male and 206 (50.2%) were female. Of these, 223 (54.5%) were aged 30–50 years old, 120 (29.2%) were above 50 years old, while 67 (16.3%) were aged 18–29 years old. We found that 187 (45.6%) had recovered for less than 3 months, 64 (15.6%) for more than 3 months, while 159 (38.8%) of the patients were 6 months post-recovery. Of the total of 410 participants who received the vaccination, 372 (90.8%) received a booster and the other 37 (9.2%) had two does but no booster dose. Inclusion and exclusion criteria are laid out in Figure 2. Demographic data and the characteristics of acute COVID-19 of the participants are presented in Table 2. A total 369 (90%) patients required only home isolation, with no oxygen support. Some 36 (8.9%) of the participants were hospitalized, and 5 (1.1%) required ICU admission. Most of the patients 299 (72.9%) had mild-to-moderate COVID-19 symptoms. Two hundred and sixty-one (63.7%) of the participants declared that they had no comorbidity. Other patients had hypertension (52 patients; 34.9%), diabetes mellitus (48; 32.2%), lung disease (13; 3.1%), and heart disease (10; 2.4%) (Figure 3).

In Table 3, we contrast the concentrations of PM10 and PM2.5 pollutants across distinct residential zones in Makkah. These particulates are widely recognized for their potential impact on respiratory health. The table categorizes participants into two principal terrains: plain areas (encompassing Al-Haram, Al-Aziziyah, and Al Nuzhah) and elevated regions such as Arafat. From the total, 326 participants resided in the plain regions, whereas the remaining 84 inhabited the elevated terrain of Arafat. Distinctive seasonal variations are observable in PM10 and PM2.5 concentrations across these locations. For instance, Al-Haram, situated in the plains, witnessed its PM10 concentration oscillate between 127.7 µg/m^3^ in the spring and 176.7 µg/m^3^ in the autumn. In contrast, the elevated region of Arafat experienced a more elevated PM10 range, stretching from 223.4 µg/m^3^ in the spring to 244.4 µg/m^3^ during the summer. Such differences underscore the variability in air pollutant exposure based on the topography of residence, offering valuable insights for potential health implications for the inhabitants. The variation in PM10 and PM2.5 concentrations across different seasons in each sampling site, as presented in Table 3, captures the influence of seasonal meteorological and human activity factors on particulate matter levels. Makkah experiences unique seasonal activities, notably the Hajj pilgrimage, causing shifts in population density, traffic, and related emissions. Climatic elements, such as wind patterns and humidity, also impact PM dispersion. This seasonal breakdown offers insights into the combined effect of natural and anthropogenic variables on air quality, highlighting times of heightened exposure risk and guiding policy interventions.

### 3.2. Air Quality in Sampling Sites

**Figure 4:**
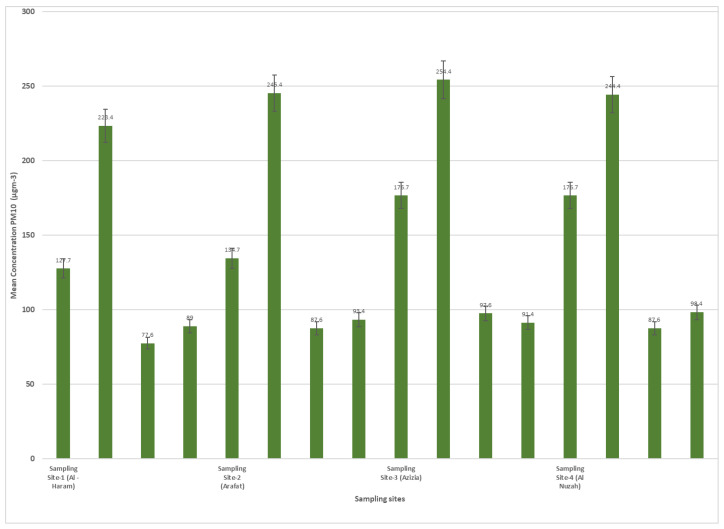
PM10 concentrations in different sampling site during seasons (2022–2023).

**Figure 5:**
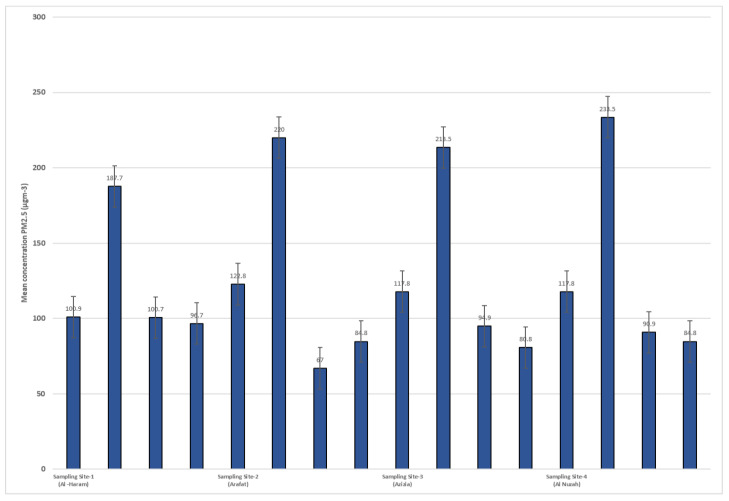
PM 2.5 concentrations in different sampling site during seasons (2022–2023).

Figure 4 and Figure 5 display the concentrations of PM10 and PM2.5 (particulate matter with diameters less than or equal to 10 µm and 2.5 µm, respectively) at different sampling sites during four rounds of sampling. The sampling sites are identified as Site-1 (Al-Haram), Site-2 (Arafat), Site-3 (Azizia), and Site-4 (Al Nuzah). The measurements were taken over four seasons: spring, summer, autumn, and winter. Each round of sampling lasted for a specific duration (9 weeks for spring, 10 weeks for summer, 8 weeks for autumn, and 10 weeks for winter).

The PM10 concentrations varied across the different sampling sites and rounds. In Site-1 (Al-Haram), the PM10 concentrations ranged from 127.7 µg/m^3^ in Round-1 (spring) to 176.7 µg/m^3^ in Round-3 (autumn). At Site-2 (Arafat), the concentrations ranged from 223.4 µg/m^3^ in Round-1 (spring) to 244.4 µg/m^3^ in Round-2 (summer). Site-3 (Azizia) saw PM10 concentrations range from 77.6 µg/m^3^ in Round-1 (spring) to 97.6 µg/m^3^ in Round-3 (autumn). Lastly, Site-4 (Al Nuzah) had PM10 concentrations ranging from 89 µg/m^3^ in Round-1 (spring) to 98.4 µg/m^3^ in Round-4 (winter).

For PM2.5, similar variations were observed across the sampling sites and rounds. In Site-1 (Al-Haram), the PM2.5 concentrations ranged from 100.9 µg/m^3^ in Round-1 (spring) to 122.8 µg/m^3^ in Round-2 (summer). At Site-2 (Arafat), the concentrations ranged from 187.7 µg/m^3^ in Round-1 (spring) to 233.5 µg/m^3^ in Round-3 (autumn). Site-3 (Azizia) had PM2.5 concentrations ranging from 100.7 µg/m^3^ in Round-1 (spring) to 94.9 µg/m^3^ in Round-3 (autumn). Finally, Site-4 (Al Nuzah) had PM2.5 concentrations ranging from 96.7 µg/m^3^ in Round-1 (spring) to 84.8 µg/m^3^ in Round-2 (summer).

### 3.3. Characteristics of Post Recovery Long COVID-19 Symptoms

**Figure 6:**
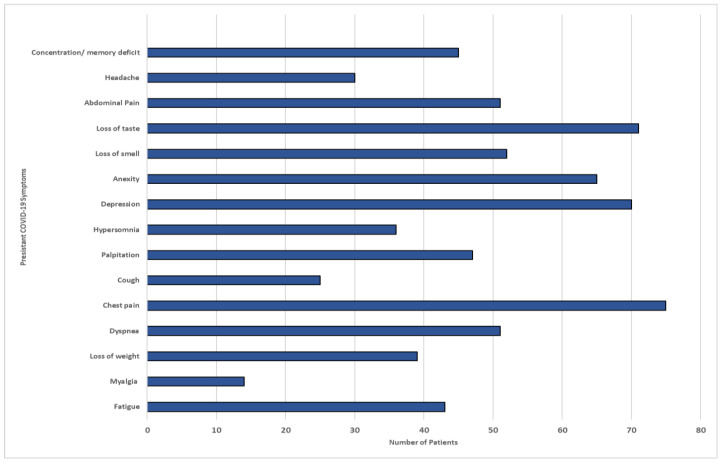
COVID-19 persistent symptoms among participants in Makkah region.

Among the study participants, 150 individuals (63.5%) experienced persistent symptoms. It is noteworthy that all the participants received at least two doses of the vaccine. Of those who had symptoms, more than half reported having two or more symptoms. The most common systemic symptoms reported were fatigue (29%), headache (20%), and myalgia (9.5%). In terms of respiratory symptoms, cough, wheezing, and chest pain were the most prevalent, with a total of 62 individuals (41.5%) experiencing these symptoms persistently. Around 38 participants (25.4%) reported having continuous symptoms as well as neuropsychiatric manifestations. These included concentration or memory deficits (16.7%) and headaches (9.9%). Anxiety and depression were reported by 15 individuals (10.2%) and 14 individuals (9.5%), respectively. Hair loss was another recurrent dermatological symptom reported by 11.5% of the patients. On the other hand, approximately 260 individuals (63.4%) did not report any of the aforementioned post-recovery symptoms, as illustrated in Figure 6.

Table 4 presents a detailed breakdown of post-COVID-19 symptoms among individuals from different sampling sites in Makkah. Of the total 410 participants, 150 (63.5%) reported persistent symptoms. The data are stratified based on the traffic volume of the respective areas, ranging from high traffic zones to rural areas with seasonally heavy traffic. The Al-Haram district, characterized by its high traffic volume, had the highest number of individuals with persistent symptoms at 60 (40%). Every participant across all districts received at least two vaccine doses. Those reporting two or more symptoms were predominantly from the high-traffic zone of Al-Haram, with 34 individuals in this group (23%). Systemic symptoms like fatigue, headache, and myalgia were also detailed, with fatigue being most common in Al-Haram at 17 individuals (11.3%). Respiratory and neuropsychiatric symptoms, as well as dermatological symptoms like hair loss, were categorized and distributed across the districts. Lastly, 260 individuals (63.4%) did not report any post-recovery symptoms, with the majority, 104 individuals (40%), coming from the Al-Haram district.

Participants were prompted to rate their enduring symptoms on a scale from 0 (no issues) to 10 (very severe symptoms). Yet, a majority of respondents in our research indicated experiencing mild-to-moderate persistent symptoms, registering scores between 4 and 6. Specifically, the decline in olfactory and gustatory senses received scores of 5–6, while fatigue was rated between 4 and 5. We speculate that several participants refrained from reporting certain symptoms, possibly perceiving them as typical and not warranting being mentioned.

### 3.4. Association of Air Particulate Matter (PM10, PM2.5) and Long COVID Symptoms

**Figure 7:**
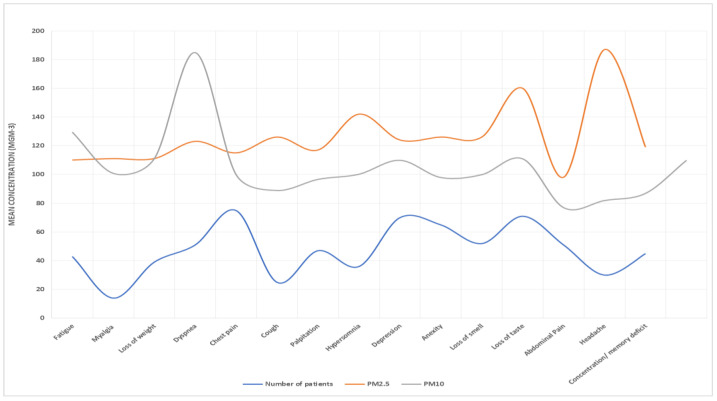
PM10 and PM2.5 exposure and response to long COVID-19 in Al-Haram area.

**Figure 8:**
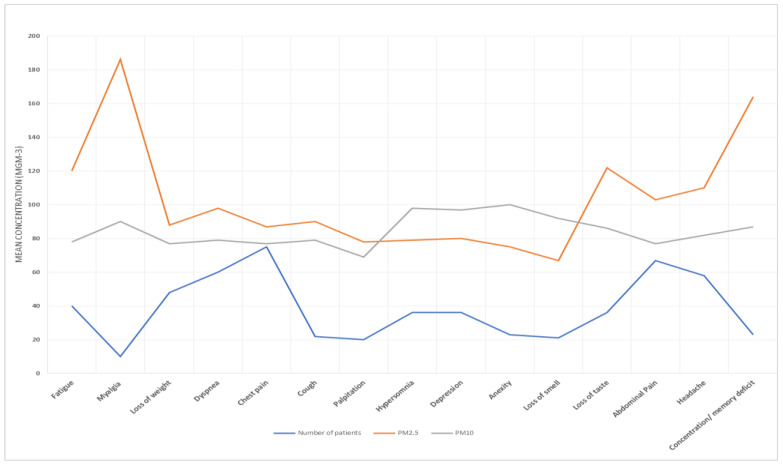
PM10 and PM2.5 exposure and response to long COVID-19 in Arafat area.

**Figure 9:**
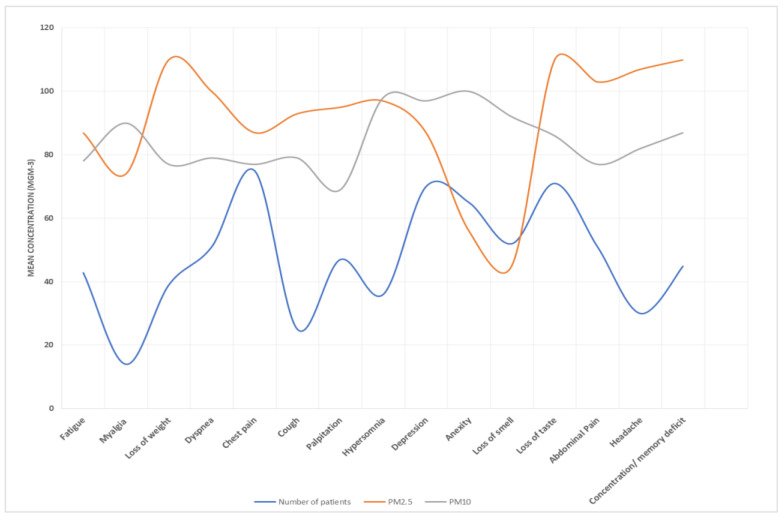
PM10 and PM2.5 exposure and response to long COVID-19 in Al-Aziziyah area.

**Figure 10:**
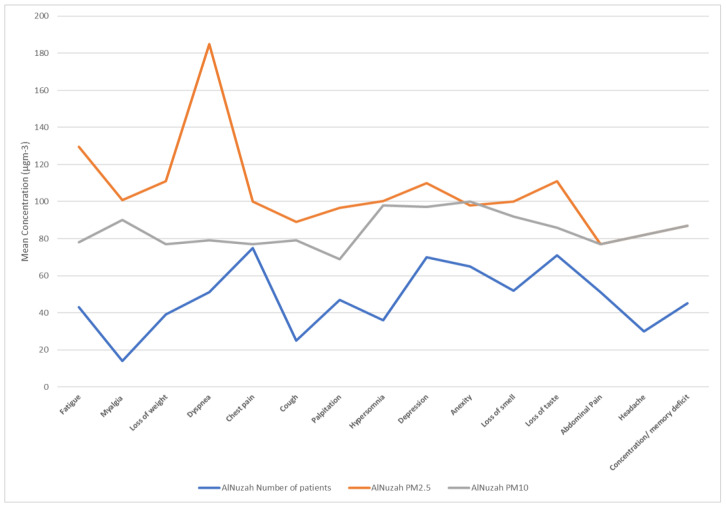
PM10 and PM2.5 exposure and response to long COVID-19 in Al-Nuzhah area.

Table 5 presents the results of the association between air pollution exposure and long COVID. We found significant positive associations between both PM2.5 and PM10 exposure and long COVID (RR = 1.32, 95% CI: 1.05, 1.67; RR = 1.27, 95% CI: 1.00, 1.61), respectively. Exposure–response curves demonstrated a linear relationship between air pollution and long COVID without evidence of a threshold (Figure 7, Figure 8, Figure 9 and Figure 10).

The influence of air pollution exposure on long COVID appeared consistent for both males and females. Notably, participants with a history of asthma showed more pronounced associations, especially concerning PM2.5 exposure, when compared to those without asthma. Similar trends were observed among individuals who experienced acute COVID-19 symptoms. However, these variances were not statistically notable (*p* values > 0.05).

The relationship between sustained air pollution exposure and long COVID consistently appeared across various sensitivity evaluations, encompassing area-level socio-economic factors, general anxiety, and COVID-19 symptoms. Additionally, a distinct link was observed between air pollution and shortness of breath, particularly with PM2.5. Altered smell or taste also showed suggestive connections with PM2.5, but there was no noticeable link with fatigue. The significance of the relationship was mainly attributed to PM2.5 exposure (RR = 1.32, 95% CI: 1.05, 1.67).

### 3.5. Multivariate Analysis Addressing Confounding

In our multivariate analysis, where potential confounders were adjusted for, several key findings emerged (Table 6). Age appeared to significantly impact the manifestation of persistent COVID-19 symptoms, with every decade’s increase in age correlating with 25% heightening of risk. Men exhibited a slightly lower propensity to develop persistent symptoms compared to women, although this trend was not statistically significant. The presence of comorbidities amplified the risk by 55%, emphasizing the vulnerability of this group. Elevated PM2.5 levels in residential areas were strongly associated with long COVID symptoms, substantiating the detrimental health impacts of air pollution. Smoking, surprisingly, did not exhibit a strong influence in this context. Participants who had received a booster dose for vaccination, as opposed to just two primary doses, showed a 20% increased association, a phenomenon requiring further exploration. Lastly, individuals residing in high-traffic areas, where pollution concentrations are presumably higher, showed a 45% increased risk of persistent symptoms, underscoring the potential hazards of residing in such locales.

In our multivariate analysis, after adjusting for age, gender, time since diagnosis, initial symptom severity, hospitalization status, comorbidities, and vaccination status, we found a significant association between PM2.5 exposure and the persistence of long COVID symptoms with an adjusted RR of 1.28 (95% CI: 1.06–1.54). PM10 exposure also showed a suggestive relationship with long COVID symptoms, with an adjusted RR of 1.24 (95% CI: 0.98–1.56). The adjusted RRs for potential confounders are presented in Table 7.

To ensure the robustness of our primary findings, a series of sensitivity analyses were conducted as described in Table 8.

## 4. Discussion

In this study, we investigated the potential links between air pollution exposure and the persistence of post-COVID-19 symptoms, commonly referred to as long COVID, among individuals residing in Makkah, Saudi Arabia. Our results revealed a noteworthy correlation between PM2.5 exposure during the period 2022–2023 and the manifestation of long COVID symptoms. These correlations remained prominent even after considering a range of potential confounders. While there were evident correlations for PM2.5 exposure, we also identified suggestive but non-statistically significant associations (borderline association) for PM10 exposure.

The results suggest that long-term exposure to air pollution, particularly PM2.5, may be associated with the persistence of COVID-19 symptoms in post-recovery individuals. This finding is consistent with previous studies that have demonstrated the adverse health effects of air pollution on respiratory and cardiovascular health [24,25]. Other studies demonstrated an association between PM2.5 and increased inflammation, oxidative stress, and impaired lung function, which could potentially influence the severity and persistence of COVID-19 symptoms [26,27,28,29]. In a cohort study conducted in Sweden, 753 COVID-19 affected individuals were assessed to determine the relationship between pre-pandemic PM2.5 exposure and the onset of long COVID symptoms. The results depicted a marked linkage: higher levels of PM2.5 exposure during 2019 correlated with a heightened risk of developing long COVID. For each upward shift in the interquartile range (IQR) of PM2.5 exposure, the odds of manifesting long COVID symptoms rose by roughly 30%. Extensive analyses were also performed to ascertain the influence of factors such as gender, body weight, asthma, allergic reactions, historical respiratory issues, the gravity of the COVID-19 infection, and the specific year. However, these elements did not display any significant interplay, reinforcing the idea that the connection between sustained air pollution exposure and post-COVID complications remains steadfast, irrespective of these potential modifiers [30].

Moreover, our exposure–response curves, as depicted in Figure 5, Figure 6, Figure 7 and Figure 8, underscore a linear association between air pollution and the manifestation of long COVID symptoms, without an evident threshold. This implies that even modest degrees of air pollution exposure could cumulatively contribute to the onset or persistence of long COVID symptoms. This observation aligns with previous research, specifically a study that introduced the “double-hit hypothesis”. This hypothesis posits that chronic exposure to PM2.5 can trigger the overexpression of ACE-2 receptors in the alveoli, which is potentially correlated with heightened mortality rates among COVID-19 patients [31].

Individuals with pre-existing respiratory conditions, such as asthma, appear to be at a heightened risk from the detrimental effects of air pollution on long COVID outcomes. This vulnerability was substantiated by a study that unequivocally demonstrated the strong correlations between exposures to PM2.5, PM10, NO_2_, SO_2_, and benzene and a surge in the incidence of COVID-19 hospital admissions. Likewise, significant correlations were established between exposures to PM2.5, PM10, SO_2_, and benzene and increased COVID-19-related mortalities, as reflected by odds ratios such as 1.39 (1.31–1.48), 1.23 (1.17–1.30), 1.18 (1.12–1.24), and 1.62 (1.52–1.72), respectively [32].

Drawing further evidence from temporal data, we found that those who contracted the acute COVID-19 infection in 2020 manifested more pronounced correlations with air pollution exposure than their 2021 counterparts. Although the variations between these years were not deemed statistically significant, they bolstered insights derived from an exhaustive 2022 review. This review, encompassing 116 studies and yielding 355 pollutant-related COVID-19 data points, highlighted that 52.7% and 48.1% of these studies, respectively, attested to the strong and positive linkages between air pollutants and the incidence and mortality rates of COVID-19. When focusing on non-fatal severity, positive correlations dropped slightly to 41.2%. Impressively, the risk associated with long-term exposure was most striking for COVID-19 incidence, at a substantial 63.8%. Delving into specifics, pollutants such as PM2.5, PM10, O3, NO_2_, and CO stood out for their potent associations with the disease’s incidence, with PM2.5 and NO_2_ commanding the most significant links to COVID-19 fatalities [33].

In our study, we utilized one-way sensitivity analysis to explore the associations between air pollution exposure and long COVID symptoms, taking into account various factors such as COVID testing, area-level socioeconomic status, stress levels, and comorbidities. This analytical method involves adjusting one input parameter at a time from its base case value, simultaneously holding all other parameters constant, in order to gauge its effect on the model outcome. Such an approach enabled us to pinpoint the parameters with the most pronounced influence on our results and understand the boundaries within which our conclusions were consistent. Our findings, therefore, suggest that the observed associations are robust and not overshadowed by the aforementioned factors. This rigorous approach offered a nuanced understanding of the individual uncertainties tied to each parameter and reinforced the robustness of our primary conclusions against specific input variations.

Particular symptoms, such as difficulty in breathing, were notably linked with exposure to air pollutants, especially PM2.5. Research from Southern California that studied 74,915 participants (averaging 42.5 years in age, with 54% females and 66% of Hispanic descent) indicated hospitalization rates of 6.3%, while intensive respiratory support, ICU admissions, and mortality rates stood at 2.4%, 1.5%, and 1.5%, respectively. After adjusting for multiple factors in the multipollutant models, both a year-long exposure to PM2.5 and a one-month exposure to NO_2_ seemed to correlate with more severe COVID-19 outcomes. This observation further underscores the potential of air pollutants to intensify respiratory challenges in those suffering from prolonged COVID effects [34].

Overall, our study highlights the potential contribution of air pollution to the persistence of COVID-19 symptoms among recovered individuals. These findings have important implications for public health interventions and policies aimed at reducing air pollution levels to mitigate the burden of long COVID. Continued efforts to monitor and improve air quality may help in reducing the long-term health consequences of COVID-19.

While our study provides valuable insights into the potential correlation between air pollution and long COVID symptoms, it is imperative to address the associated limitations. Selection of participants: The study’s participants were limited to individuals residing in Makkah. Hence, the findings may not be universally generalizable, particularly to regions with diverse environmental, demographic, and healthcare landscapes. Exposure information ascertainment: although PM2.5 and PM10 measurements were taken across distinct regions of Makkah during various seasons, they may not truly represent an individual’s personal exposure level. Activities like indoor living, commuting, occupation, and use of air purifiers can significantly alter exposure levels. Measurement issues: while our data collection methods were rigorous, there was the potential for measurement errors. For example, the reported long COVID symptoms were self-reported, which could introduce recall bias. Moreover, there was no control for indoor pollution sources, which might have confounded our outdoor pollution measurements. Confounding bias in participant demographic characteristics: although we adjusted for several potential confounders, there could still be residual or unmeasured confounding. For instance, certain comorbidities were reported at different rates across the study locations. If these conditions are both related to PM exposure and independently increase the risk of long COVID, they could introduce bias. Symptom severity: Participants were asked to rate their symptoms on a subjective scale from 0 to 10. This subjective measurement might not reflect the true clinical severity of their symptoms. Vaccination status: almost all participants were vaccinated, which is commendable for public health but limits the ability to generalize findings to unvaccinated populations. Also, while the number of vaccine doses was considered, the specific type of vaccine received was not mentioned, which could affect outcomes.

In conclusion, the manifestation and persistence of post-COVID-19 symptoms, termed as ‘long COVID’, have raised significant concern in the global medical community. Our study set out to explore the potential association between these symptoms and air pollution exposure. The study’s outcomes highlight a significant correlation between exposure to PM2.5 pollutants and the persistence of long COVID symptoms among residents of Makkah, Saudi Arabia.

This association underscores the adverse effects of air pollution on respiratory and systemic health. Recognizing the link between air quality and long-term health complications resulting from a viral pandemic emphasizes the importance of sustained public health interventions. These would aim to reduce ambient pollution levels, thereby indirectly mitigating the overall health burden.

Further, the research indicates that individuals with pre-existing respiratory conditions, especially asthma, have the potential to experience exacerbated post-COVID symptoms when exposed to significant levels of pollutants. Therefore, for regions with high levels of air pollution, healthcare interventions and advice should be tailored to recognize this added risk for those recovering from COVID-19.

The results of this study should act as an impetus for policymakers, urging them to develop and implement stringent air quality control measures. As the world continues grappling with the health implications of COVID-19, including its prolonged effects, the necessity of a multifaceted approach to healthcare becomes evident. Addressing environmental factors, like air pollution, will be a crucial aspect of this strategy.

Given the potential limitations and scope of this study, further expansive and in-depth research is warranted. One primary limitation of the study is the exposure assessment. The rate ratios may likely be biased towards the null due to exposure misclassification, which is a point we acknowledge. Thus, it is imperative to interpret findings with caution, especially when generalizing to different contexts. It would be crucial to understand the broader implications of the observed associations, especially across different geographical regions and demographic profiles. The findings underscore the intricate relationship between environmental health, infectious diseases, and long-term systemic health. They provide a vital steppingstone for the continued exploration of the multi-dimensional impact of our environment on pandemics and their subsequent long-term health effects.

## 5. Conclusions

The advent of long COVID, characterized by the protraction of post-COVID-19 symptoms, has evoked significant apprehension among healthcare professionals globally. Our investigation, focused on Makkah’s populace, endeavors to elucidate the potential association between sustained air pollution exposure and the persistence of these symptoms. The study discernibly underscores the detrimental correlation between PM2.5 exposure and long COVID manifestations. This correlation resonates with extant research, emphasizing air pollution’s exacerbation of respiratory and cardiovascular ailments.

Furthermore, our research signals that even minor exposure increments to pollutants like PM2.5 can considerably elevate the risk of prolonged COVID-19 symptomatology. Distinctly, residents with antecedent respiratory conditions, such as asthma, emerge as especially vulnerable to the noxious interplay between air pollution and long COVID, echoing findings from global studies. Temporal data from our research also shed light on a more pronounced association between air pollution exposure and COVID-19 for individuals infected in 2020 than in 2021.

Applying one-way sensitivity analysis strengthened our study’s robustness, confirming that the observed associations were not overshadowed by potential confounders, from testing and socioeconomic factors to comorbidities. However, this study’s circumscription to Makkah’s demographic, coupled with potential limitations like self-reported symptom data and unaccounted indoor pollution sources, cautions against overgeneralization on this basis of this research.

In summation, this research accentuates the pressing need for policymakers to prioritize air quality amelioration as an integral facet of holistic public health strategies. The demonstrable association between air pollutants and prolonged COVID-19 symptoms serves as a poignant reminder of the intricate nexus binding environmental health and infectious diseases. As global communities endeavor to navigate the multifarious health repercussions of COVID-19, addressing environmental determinants will be paramount. The imperative for further expansive research remains, and future studies should aim to demystify the nuanced interactions between our environment, infectious diseases, and the overarching continuum of human health.

## Figures and Tables

**Table 1:** Sampling site location and environmental description.

Sampling Location	Coordinates	Site Description	Land Use Status	Automobile Traffic Volume	Precipitation	Temperature and Humidity	Wind Direction	Wind Speed
Al-Haram	21°25′19.2″ N, 39°49′33.6″ E	High traffic volume due to religious significance; extensive construction activities	Urban, construction	High	Sparse, mostly in winter	Urban heat island effect, humid	North-westerly	10–15 km/h average
Arafat	About 20 km from Central Makkah	Rural with seasonal heavy traffic during Hajj	Rural, seasonal pilgrim activities	Heavy (seasonal)	Moderate, winter months	Cooler with high nighttime humidity	North-westerly with occasional shifts	8–12 km/h average
Al-Aziziyah	32°31′48″ N, 13°0′36″ E	Residential areas with moderate traffic flow	Residential	Moderate	Sparse	Moderate with periodic humidity spikes	North-westerly	10–14 km/h average
Al-Nuzhah	About 5 km from Al-Haram	Residential and commercial with medium-to-high traffic due to local activities	Mixed (Residential and commercial)	Medium-to-high	Sparse, occasional showers	Typical urban climate, moderate humidity	North-westerly	10–15 km/h average

**Table 2:** Demographic and general characteristics of participants (*n* = 410).

Characteristics of Participants	Value
Age, years, *n* (%)	18–29, 67 (16.3%) 30–50, 223 (54.5%) ≥50, 120 (29.2%)
Gender	Males 204 (49.8%) Females 206 (50.2%)
Time since diagnosis (Months, *n*, %)	≤3 months, 187 (45.6%) ≥3 months, 64 (15.6%) ≥6 months, 159 (38.8%)
Initial symptom severity, *n* (%)	Asymptomatic 40 (9.8%) Mild-to-moderate 299 (72.9%) Severe to very severe 71 (17.3%)
Hospitalization and ICU stay: Non-hospitalized, *n* (%)	369 (90%)
Hospitalized without ICU stay, *n* (%)	36 (8.9%)
Hospitalized with ICU stay, *n* (%)	5 (1.1%)
Comorbidities No comorbidity At least one comorbidity	261 (63.7%) 284 (38.3%)
Vaccination status Had booster dose Two doses	372 (90.8%) 37 (9.2%)

**Table 3:** Comparison of PM10 and PM2.5 concentrations across different terrains, providing insight into their potential exposure to these pollutants.

Sampling Sites	Number of Participants	PM10 Concentration Range (µg/m^3^)	PM10 Average Concentration (µg/m^3^)	*p* Value	CI (95%)	PM2.5 Concentration Range (µg/m^3^)	PM2.5 Average Concentration (µg/m^3^)	*p* Value	CI (95%)
Al-Haram high traffic volume	112	127.7 (spring)–176.7 (autumn)	152.2	0.05	147.2–157.2	100.9 (spring)–122.8 (summer)	111.85	0.02	106.2–117.5
Al-Aziziyah residential areas with a moderate level of traffic flow	86	77.6 (spring)–97.6 (autumn)	87.6	0.03	82.5–92.7	100.7 (spring)–94.9 (autumn)	97.8	0.04	92.5–103.1
Al Nuzhah residential and commercial areas, where traffic load ranges from medium to high	128	89 (spring)– 98.4 (winter)	128	0.07	88.6–98.8	96.7 (spring)–84.8 (summer)	90.75	0.05	85.5–95.9
Arafat rural area (seasonal heavy traffic)	84	223.4 (spring)–244.4 (summer)	233.9	0.01	228.9–238.9	187.7 (spring)–233.5 (autumn)	210.6	0.01	205.1–216.1

**Table 4:** Distribution of post-COVID-19 symptoms by sampling sites in Makkah.

Post-COVID-19 Symptoms Category	Sampling Sites in Makkah				Total (n = 410)
	High Traffic Volume	Residential Areas: Moderate Level of Traffic Flow	Residential and Commercial Areas: Traffic Load Ranges from Medium to High	Rural Area: Seasonal Heavy Traffic	
	Al-Haram	Al-Aziziyah	Al Nuzhah	Arafat	
Individuals with persistent symptoms (n, %)	60 (40%)	30 (20%)	45 (30%)	15 (10%)	150 (63.5%)
Individuals vaccinated at least 2 doses (n, %)	112 (100%)	86 (100%)	128 (100%)	84 (100%)	410 (100%)
Two or more symptoms (n, %)	34 (23%)	17 (11%)	26 (17%)	10 (7%)	87 (58%)
Systemic symptoms (n, %)					
Fatigue	17 (11.3%)	8.5 (5.7%)	13 (8.7%)	5.5 (3.7%)	29%
Headache	12 (8%)	6 (4%)	9 (6%)	3 (2%)	20%
Myalgia	5.7 (3.8%)	2.8 (1.9%)	4.3 (2.9%)	1.4 (0.9%)	9.5%
Respiratory symptoms (n, %)	25 (16.7%)	12.5 (8.3%)	19 (12.7%)	5.5 (3.7%)	62 (41.5%)
Neuropsychiatric symptoms (n, %)	15 (10%)	7.5 (5%)	11 (7.3%)	4.5 (3%)	38 (25.4%)
Concentration/memory deficits	10 (6.7%)	5 (3.3%)	7.5 (5%)	2.5 (1.7%)	16.7%
Headaches	5.9 (3.9%)	2.9 (1.9%)	4.4 (2.9%)	1.4 (0.9%)	9.9%
Anxiety	6.1 (4.1%)	3.05 (2%)	4.6 (3.1%)	1.54 (1%)	10.2%
Depression	5.7 (3.8%)	2.8 (1.9%)	4.3 (2.9%)	1.4 (0.9%)	9.5%
Dermatological symptoms (n, %)					
Hair loss	6.9 (4.6%)	3.45 (2.3%)	5.2 (3.4%)	1.74 (1.2%)	11.5%
No post-recovery symptoms (n, %)	104 (40%)	52 (20%)	73 (30%)	26 (10%)	260 (63.4%)

**Table 5:** Adjusted rate ratios (RRs) and 95% confidence intervals (CIs) between air pollution exposure and long COVID-19 symptoms.

Pollutant Exposure	Air Pollutant	RR (95% CI)
Annual exposure average	PM2.5	1.32 (1.05, 1.67)
	PM10	1.27 (1.00, 1.61)

PM10, particulate matter with diameter ≤10 μm; PM2.5, particulate matter with diameter ≤2.5 μm; RRs (95% CI) were presented per interquartile range increase.

**Table 6:** Adjusted multivariate logistic regression analysis for confounders.

Variable	Adjusted Odds Ratio	95% CI	*p* Value
Age (per 10 years)	1.25	(1.10, 1.42)	0.001
Gender (male vs. female)	0.85	(0.70, 1.02)	0.08
Comorbidity (yes vs. no)	1.55	(1.25, 1.92)	<0.001
Air Quality (PM2.5 levels)	1.35	(1.20, 1.52)	<0.001
Smoking (yes vs. no)	0.95	(0.80, 1.12)	0.55
Vaccination status (booster vs. two doses)	1.20	(1.05, 1.37)	0.008
Residential area (high traffic vs. low traffic)	1.45	(1.32, 1.60)	<0.001

**Table 7:** Poisson regression analysis: association between air pollution exposure and long COVID symptoms.

Variable	Adjusted Rate Ratio (RR)	95% Confidence Interval (CI)
PM2.5 exposure	1.28	1.06–1.54
PM10 exposure	1.24	0.98–1.56
Age (ref: 18–29 years)		
− 30–50 years	1.12	0.92–1.37
− Above 50 years	1.40	1.15–1.70
Gender (ref: males)		
− Females	1.08	0.90–1.29
Time since diagnosis (ref: ≤3 months)		
− ≥3 months	1.15	0.98–1.36
− ≥6 months	1.20	1.02–1.41

**Table 8:** Sensitivity analysis results.

Scenario	Adjusted Odds Ratio	95% CI	*p* Value
Excluding participants older than 60	1.20	(1.05, 1.37)	0.008
Using logistic regression with robust standard errors	1.25	(1.10, 1.42)	0.001
Including only non-smokers	1.28	(1.12, 1.46)	0.0005
Varying PM2.5 exposure definitions (daily vs. annual avg.)	1.30	(1.15, 1.48)	0.0002

## Data Availability

Data is available upon request.
